# A War-Time Christmas in Hospitals

**Published:** 1917-12-08

**Authors:** 


### A WAR-TIME CHRISTMAS IN HOSPITALS.

It is extraordinary what a number of cold hearts seem to exist in human bodies belonging to people, who have not suffered, personally or directly, from the war. This type seems to rejoice in snubbing the Christmas festival. The aim is to reduce everything concerning it to such unworthy and miserable proportions as to create a day of exasperating irritations to every occupant of a hospital, in substitution for the joyous, uplifting, wholesome centre of blessed memories, which some of the best and largest of our hospitals have made it, by a great outpouring of extra personal service, voluntarily rendered, on the part of the sisters and nurses especially, and of the students and medical residents too. We have declared from conviction for decades of years that, to the earnest-minded and some of the best members of our race, a hospital of the type just mentioned on Christmas Day, is a place to cultivate and visit every year without fail.

But this terrible war has smitten our hospitals by taking away from them many of the most active and valued members of their staffs, thereby reducing materially the number of workers within their walls. It has also largely increased the number of patients who have to be housed and cared for owing to the presence of wounded warriors from the fighting line. These conditions may render it difficult or impossible to keep Christmas Day on the old lines, but the spirit of Christmas everywhere throughout our hospitals must be upheld and cultivated. The worst thing Scrooge could say about Christmas the advocates of substituted plum-pudding and the cultivation of dulness will not admit. Here are Scrooge's words: "If I could work my will every idiot, who goes about with 'Merry Christmas' on his lips, should be boiled with his own pudding and buried with a stake of holly through his heart." The opponents of the English Christmas urge they only want the abolition of the things that are unreasonable and unnecessary. These things embrace the strict reduction of all kinds of food supplied on Christmas Day, and the abolition of "the false generosity of a genuine plum-"pudding and Christmas fare." Now cheerfulness in the sick is the high road to recovery. To forbid all decorations, and abolish all amusements, entertainments, music and brightness from the Christmas festival in our hospitals, to change the dinner to an untempting and stinted diet, could not fail to eliminate cheerfulness, to depress the inmates of every hospital which so set itself to destroy Christmas joy, and to make this day the dullest, and in memory the most horrible in the whole year. Is there one kill-joy of the cold-heart type, if he thus had his way, who would have the courage to face the patients and the workers in such a hospital of his desires? We trow not, for brightness, increased life, good fellowship, loving care and personal service are, in the fourth year of the war, more needed, and will be more welcome, than ever, in every British hospital worthy of the name.

There are some things, however, which should be abolished. Hospital managers and the matrons must make it their business this year to prevent the possibility that, in any hospital the sisters have to give out of their own pocket £5 or more for the decoration of their wards and the provision of a handsome tea. Neither sisters nor nurses should be permitted to pay anything out of their own pockets in this connection, much less to subscribe to a present or testimonial for some or all the members of the staff at this season. Every chairman in every hospital should make it his business to secure the abolition of these practices wherever they exist. Miss Rosa Smith, the secretary of the J. S. Morgan Benevolent Fund, insists that the sisters in some infirmaries are the worst sufferers. Let Christmas 1917 see the end of such suffering, and the abolition of presents for everyone from the matron downwards. These changes will be true economy of the very best.

The members of hospital committees, and interested governors of institutions where the practice does not prevail, should mark Christmas Day this year by providing in advance all the expenses necessary to give everybody in each of our hospitals, a worthy, inspiriting, uplifting, joyous, happy Christmas Day. Then Scrooge and the cold-hearted kill-joys would be silenced, and personal service should perfect the arrangements and make them worthy of the day and Christ's coming. We are sending Christmas fare and greetings to our fighting men of both Services. At home our hospitals contain thousands of their colleagues who are wounded and suffering, and they too must have a joyous Christmas, everybody will agree. So also must the civilian patients, for have not they or members of their families devoted their energies, with patriotic and continuous zeal, to the supply of munitions, and everything, without which, our fighting men could not have rendered the splendid services to the nation, which have made us all proud to be members of the British Empire?

